# Species Separation within, and Preliminary Phylogeny for, the Leafhopper Genus *Anoscopus* with Particular Reference to the Putative British Endemic *Anoscopus duffieldi* (Hemiptera: Cicadellidae)

**DOI:** 10.3390/insects11110799

**Published:** 2020-11-13

**Authors:** Joanna Redihough, Isa-Rita M. Russo, Alan J. A. Stewart, Igor Malenovský, Jennifer E. Stockdale, Rosemary J. Moorhouse-Gann, Michael R. Wilson, William O. C. Symondson

**Affiliations:** 1 Cardiff School of Biosciences, Sir Martin Evans Building, Museum Avenue, Cardiff CF10 3AX, UK; jredihough@gmail.com (J.R.); russoim@cardiff.ac.uk (I.-R.M.R.); jen.stockdale@nottingham.ac.uk (J.E.S.); rosemarym.gann@gmail.com (R.J.M.-G.); 2 School of Life Sciences, University of Sussex, Brighton BN1 9QG, East Sussex, UK; a.j.a.stewart@sussex.ac.uk; 3 Department of Botany and Zoology, Faculty of Science, Masaryk University, CZ-611 37 Brno, Czech Republic; malenovsky@sci.muni.cz; 4 Department of Natural Sciences, National Museum of Wales, Cardiff CF10 3NP, UK; michael.wilson@museumwales.ac.uk

**Keywords:** *Anoscopus duffieldi*, Aphrodinae, Cicadellidae, endemic species, genetic distance, molecular separation

## Abstract

**Simple Summary:**

*Anoscopus* leafhoppers are a group of plant-feeding bugs that can be found in a range of grassland habitats. There are seven recognized species in the UK, some of which are difficult to tell apart. One species, *Anoscopus duffieldi*, has only been found at a single site, an RSPB (Royal Society for the Protection of Birds) reserve at Dungeness in Kent. As *Anoscopus* leafhoppers can be quite variable in colour and pattern, and in the structure of their genitalia, our aim was to establish, using DNA, whether this ‘species’ is unique or whether it is simply a variant of one of the other species. If it is unique, found nowhere else, it should be afforded special protection. Samples of all UK species, as well as another from the Czech Republic, were collected from the field, and two genes were examined. The DNA sequences showed that three species, *A. duffieldi*, *Anoscopus albifrons* and *Anoscopus limicola* were so closely related that they should probably be considered a single species. However, *A. duffieldi* are distinctive in that they live only in one area of vegetated shingle. We suggest that, until other evidence is forthcoming, *A. duffieldi* could be considered a locally adapted subspecies of scientific interest.

**Abstract:**

The subfamily Aphrodinae (Hemiptera: Cicadellidae) contains ~33 species in Europe within four genera. Species in two genera in particular, *Aphrodes* and *Anoscopus*, have proved to be difficult to distinguish morphologically. Our aim was to determine the status of the putative species *Anoscopus duffieldi*, found only on the RSPB Nature Reserve at Dungeness, Kent, a possible rare UK endemic. DNA from samples of all seven UK *Anoscopus* species (plus *Anoscopus* *alpinus* from the Czech Republic) were sequenced using parts of the mitochondrial cytochrome oxidase I and 16S rRNA genes. Bayesian inference phylogenies were created. Specimens of each species clustered into monophyletic groups, except for *Anoscopus* *albifrons*, *A. duffieldi* and *Anoscopus* *limicola*. Two *A. albifrons* specimens grouped with *A. duffieldi* repeatedly with strong support, and the remaining *A. albifrons* clustered within *A. limicola*. Genetic distances suggest that *A. albifrons* and *A. limicola* are a single interbreeding population (0% divergence), while *A. albifrons* and *A. duffieldi* diverged by only 0.28%. Shared haplotypes between *A. albifrons*, *A. limicola* and *A. duffieldi* strongly suggest interbreeding, although misidentification may also explain these topologies. However, all *A. duffieldi* clustered together in the trees. A conservative approach might be to treat *A. duffieldi*, until other evidence is forthcoming, as a possible endemic subspecies.

## 1. Introduction

Conventional methods of species identification and separation rely on morphological features to distinguish taxa [1]. Erroneous identification can undermine taxonomy, ecological research, conservation efforts and ecosystem management [2,3,4]. Serious problems can arise when type specimens are involved [5]. Differences between morphologically similar species, and their phylogenetic relationships, can often be resolved using genetic evidence [6,7,8,9].

Auchenorrhyncha are within the fifth most diverse insect order, Hemiptera [10,11], and comprise ~43,000 species worldwide, including leafhoppers, planthoppers, treehoppers, froghoppers (spittlebugs) and cicadas [12,13]. These herbivorous insects variously feed on xylem or phloem sap or mesophyll contents [14]. Many leafhoppers are plant pathogen vectors [15,16] or are studied as part of conservation efforts [14,17] or evaluation of community structure [18]. Species separation within many leafhopper genera is seriously understudied and hampered by the presence of morphologically cryptic species and biotypes.

The subfamily Aphrodinae (Hemiptera: Cicadellidae) contains 33 species in Europe within four genera: *Stroggylocephalus* Flor, 1861; *Planaphrodes* Hamilton, 1975; *Aphrodes* Curtis, 1833; and *Anoscopus* Kirschbaum, 1868 [19,20], of which 15 species and all genera occur in the UK [21]. Species in *Aphrodes*, *Planaphrodes* and *Anoscopus* are morphologically similar, to the extent that all *Anoscopus* were previously regarded as *Aphrodes* [22,23,24,25]. The current split between *Anoscopus* and *Aphrodes* dates back to Hamilton [24]. External characters were used to distinguish between leafhopper species until the late 1930s, when the aedeagus became the primary discriminator [26]. Some *Aphrodes* and *Anoscopus* species have proved difficult to distinguish morphologically, the differences being mainly based upon the details of the male aedeagus, such as its shape and positions of spines [9,20,27]. However, these characters are subject to intraspecific variability and interspecific overlap, and females and nymphs cannot be separated reliably [9]. More accurate morphological identification of male *Aphrodes* and *Anoscopus* is generally possible using a combination of aedeagus and external morphometric measurements [9,20,28].

A possibly new species of *Aphrodes* was reported by Duffield [29] from Dungeness, Kent. Duffield noted banded elytra on three males, similar to *Aphrodes assimilis* (now *Anoscopus assimilis* (Signoret, 1879)), which had not been recorded in Britain at the time. Additional morphological characters of *A. assimilis*, published by Ribaut [22], supported Duffield’s initial identification. Subsequently, Duffield sent specimens to Le Quesne, who established it as a new species, *Aphrodes duffieldi* (now *Anoscopus duffieldi* (Le Quesne, 1964)), possibly confined to Kent, based upon aedeagus characters [30]. Le Quesne [23] later considered that *A. duffieldi* could be synonymous with *Anoscopus alpinus* (Wagner, 1955), with this continental species regarded as conspecific with *A. assimilis* by Nast [31], Hamilton [24] and, with question marks, Remane and Fröhlich [27]. Guglielmino and Buckle [20] treated *A. assimilis* and *A. alpinus* as separate species, based on differences in the forewing shape, colour and aedeagus size and structure. They also studied the morphology of nine male specimens of *A. duffieldi* from the type locality and noticed a large variability in the aedeagus morphology and external similarity to another species, *A. albifrons* (Linnaeus, 1758), suggesting that *A. duffieldi* specimens may represent hybrids between *A. albifrons* and *A. alpinus* or *A. assimilis*, but concluded that the problem needed further research.

Britain has very few endemic taxa, and therefore species (and subspecies) that are found to be endemic are often given high levels of protection (e.g., designated Sites of Special Scientific Interest) [32]. The present study independently tests the findings of previous morphologically-based work by using molecular data. Our aim was to separate species of *Anoscopus* by analysis of DNA sequences in order to resolve the status of *A. duffieldi* at its only known location in the UK and, as far as is known, the world [21] and some other taxonomic uncertainties within the *Anoscopus* genus. Accurate species separation is an essential precursor to meaningful ecological research and conservation planning in this genus.

## 2. Materials and Methods

### 2.1. Specimen Collection

**Figure 1:**
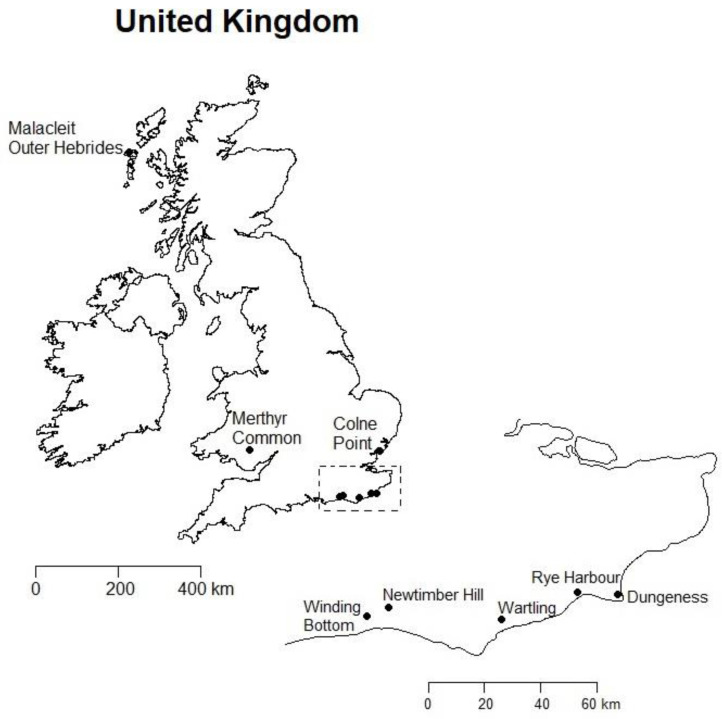
United Kingdom locations where *Anoscopus* were sampled (site details given in Table 1) with enlarged inset for SE England. Scale bars indicate distances in kilometers.

*Anoscopus* specimens from the UK were mostly collected by suction sampling between 2011 and 2015 (Figure 1). Samples of *A. alpinus* were acquired in the Czech Republic in 2015. Details of the collection sites and preservation methods are described in Table 1. The material was initially identified based on morphology using the keys by Le Quesne [23], Biedermann and Niedringhaus [25] and Wilson et al. [21]. *Anoscopus* specimens from Dungeness were attributed to *A. duffieldi* or *A. albifrons* based on the aedeagal characters used by Le Quesne [23] and Guglielmino and Buckle [20], although the published differences between these taxa are slight and some specimens displayed characters that appeared intermediate. No other *Anoscopus* species were collected from this site. Photographs and drawings of the *Anoscopus* species have been published previously [20,25,33].

### 2.2. Choice of Molecular Markers

The mitochondrial cytochrome c oxidase subunit 1 (CO1) gene in particular has been used to resolve species-level separation and relationships in animal taxa, including insects, due to its relatively rapid mutation rate [34,35], lack of recombination and highly conserved regions for relatively easy amplification from small or degraded specimens [7,36]. As such, there is a wide range of primers designed for this region [8]. Mitochondrial ribosomal genes (e.g., 16S) can also be useful for barcoding and phylogenetics of closely related species [8] but in many taxa are more conserved than the CO1 barcoding region. Mitochondrial DNA is also suitable for calculating genetic distances within and between species [37]; however, due to maternal inheritance, hybridisation between species may occur, altering phylogenetic results. For this and other reasons, parallel nuclear gene analysis has become increasingly used to ensure correct relationships. The nuclear 28S ribosomal gene has 12 divergent domains (D1-D12) within five fragments, differing in variability [38]. Some domains have been previously used in leafhopper phylogenetic studies [15,38,39]. We initially chose therefore to target regions of the CO1, 16S and 28S genes to facilitate species separation.

### 2.3. DNA Extraction and PCR Amplification

Qiagen’s DNeasy^®^ Blood and Tissue kit (Qiagen, Hilden, Germany) was used to extract DNA from all *Anoscopus* specimens following the manufacturer’s protocol.

PCR reaction mixtures for both CO1 and 16S amplification consisted of 5 μL Multiplex master mix (Qiagen, Hilden, Germany), 3.6 μL RNase free water, 0.2 μL of each primer (10 pmol/μL) and 1 μL extracted DNA with a final volume of 10 μL. All PCRs had an initial 15 min denaturation step at 95 °C. General invertebrate primers, LCO1490 and HCO2198, targeting the mt CO1 gene [40] (Table 2), used a PCR protocol with 42 cycles as follows: 30 s at 94 °C, 90 s at 50 °C and 90 s at 72 °C and a final 10 min elongation step at 72 °C. 16S rRNA primers LR-J-12887 and LR-N-13398 [41] (Table 2) were used with 35 cycles of the following: 30 s at 94 °C, 90 s at 51 °C and 90 s at 72 °C, prior to 10 min elongation at 72 °C. Successful PCR products were purified using 1.25 μL Multicore 10X Buffer, 0.5 μL TSAP and 0.25 μL EXO1 (Thermofisher Scientific, Newport, Wales, UK) in a final volume of 2 μL with one thermal cycle of 30 min at 37 °C, 15 min at 80 °C and 5 min at 12 °C, before submission to Eurofins MWG Operon (Ebersburg, Germany) for sequencing. Amplification of nuclear 28S ribosomal DNA was also attempted using two sets of primer pairs. The first pair was originally designed by Hillis and Dixon [42] with modifications by Zahniser [43]; 28SP & 28SM2 were used to target Fragment I (D2-D3) and the second pair from Dietrich et al. [38]; 28SIIF and 28SIIR amplified Fragment II (D3-D6). PCR mixes were described as above for CO1 and 16S with thermal conditions of 15 min at 95 °C and 30 cycles of 1 min at 94 °C, 1 min at 51 °C, 2 min at 72 °C and a final elongation of 7 min at 72 °C.

### 2.4. Sequencing Analysis

All sequences obtained were confirmed to be of the mitochondrial CO1 gene, because no stop codons were found and the nucleotide sequences corresponded to the expected amino acids of the first 600 bp of the CO1 gene, and this was confirmed by a Blast search. Chromatograms were analysed using Sequencher v4.9 (Gene Codes, Ann Arbor, MI, USA.), resolving sequence ambiguities and producing consensus sequences with final lengths of 600, 420 and 1358 bp for the CO1, 16S rRNA and 28S genes, respectively. Contigs were created in Sequencher, with 28S fragments I and II separately sequenced and concatenated to generate contigs before being aligned in ClustalX v2.1 [44].

### 2.5. Phylogenetic Analyses

A likelihood ratio test as implemented in jModelTest v2.1.7 [45,46] was used to determine the best-fit model of DNA substitution under the Akaike Information Criterion (AIC). Additional parameters such as base frequencies, the shape parameter of the gamma distribution [47,48] and the proportion of invariable sites (I) were also estimated. This model was subsequently used in Bayesian Inference as implemented in MrBayes v3.2 [49] and then used to calculate distances. Four chains were run for 5 × 10^6^ generations using random starting trees and flat priors. Trees and parameters were recorded every 100th generation, and two runs were performed simultaneously. Split frequencies were compared every 100th generation, and chain convergence was evaluated in Tracer v1.6 [50]. All runs used the default heating and swap parameters. In addition, FigTree v1.4.2 [51] was used to view the Bayesian trees with posterior probabilities. Three phylogenies were produced based on the mt CO1 gene, 16S rRNA gene and a concatenated dataset, with *Aphrodes bicincta* (Schrank, 1776) as the closely related outgroup which suitably resolved the ingroup taxa. The CO1 and 28S sequences for *A. bicincta* were downloaded from GenBank (accession numbers KR042069.1 for CO1 and AF304579.1 for 28S), and an archived DNA extract was sequenced for the 16S rRNA outgroup as this sequence was not present in GenBank.

### 2.6. Population-Level Analyses

Diversity indices such as haplotype diversity (the probability that two randomly chosen sequences are different in the sample) [52] and nucleotide diversity, π (the average number of nucleotide differences per site between two sequences) [53], were calculated for each phylogenetic lineage as identified in the Bayesian tree using DnaSP version 6 [54,55]. Within- and between-group pairwise estimates of nucleotide sequence divergence were generated in MEGA v6.0 [56] (Table 3 and Table 4) by implementing a correction factor as described in Nei and Li [37].

Haplotype networks for both the CO1 and 16S rRNA genes were constructed showing the minimum mutational steps between different haplotypes using TCS (Templeton Crandall Singh network) with 95% confidence limits [57]. The haplotype networks, in conjunction with frequencies and geographic distribution of different haplotypes, were used to depict geographical and potential ancestor–descendant relationships among the identified sequences.

## 3. Results

### 3.1. Nucleotide and Haplotype Diversity

Nucleotide and haplotype diversity for species (CO1 gene) varied from 0.001–0.024 and 0.38–1.00, respectively. Nucleotide and haplotype diversity for the 16S rRNA gene was lower (values varied from 0.0009 to 0.003 and 0.11 to 0.5, respectively). These rather low nucleotide diversity values for both gene regions are indicative of shallow divergences [58]. Species *Anoscopus albiger* (Germar, 1821), *A. albifrons*, *A. alpinus* and *A. serratulae* (Fabricius, 1775) were characterised by high haplotypic diversity values, indicating the high incidence of locality-specific haplotypes (Table 4).

### 3.2. Phylogenetic Analyses

The best fit General Time Reversible (GTR + G (0.089) + I (0.449)) and Hasegawa–Kishino–Yano 85 (HKY85 + G (0.024) models of substitution were applied in all phylogenetic analyses for the CO1 and 16S rRNA genes, respectively.

**Figure 2:**
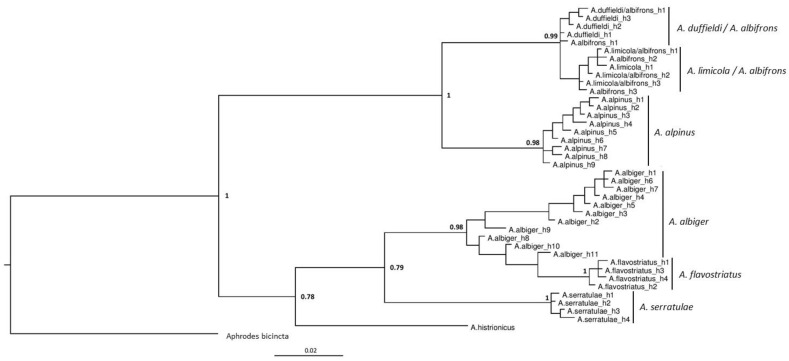
Bayesian phylogeny based on mitochondrial cytochrome c oxidase subunit 1 (CO1) DNA data. Haplotypes were identified in DnaSP v5.10.01 [55] from individual sequences, with species names separated by ‘/’ indicating shared haplotypes across two different taxa. Bayesian posterior probabilities are labelled at each node with the whole tree rooted by using the closely related *Aphrodes bicincta* as an outgroup.

**Figure 3:**
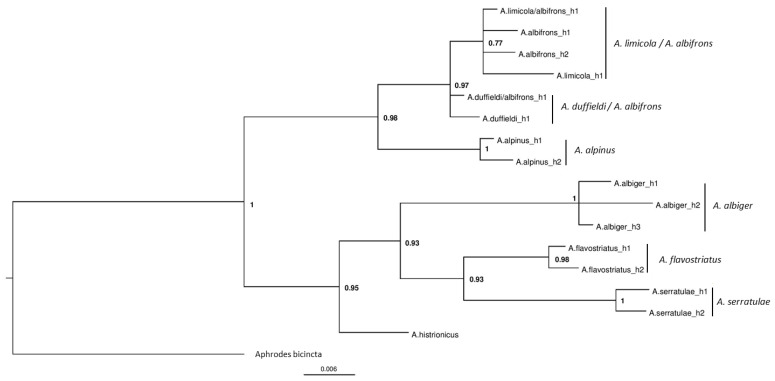
Bayesian phylogeny based on mitochondrial 16S rRNA data. Haplotypes were identified in DnaSP v5.10.01 [55] from individual sequences, with species names separated by ‘/’ indicating shared haplotypes across two different species. Bayesian posterior probabilities are labelled at each node with the whole tree rooted by using the closely related *Aphrodes bicincta* as an outgroup.

**Figure 4:**
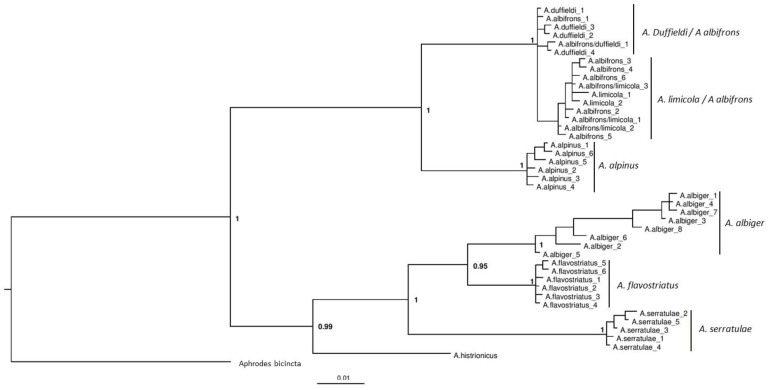
Bayesian phylogeny based on the concatenated mitochondrial CO1 and 16S rRNA data. Haplotypes were identified in DnaSP v5.10.01 [55] from individual sequences, with species names separated by ‘/’ indicating shared haplotypes across two different species. Bayesian posterior probabilities are labelled at each node with the whole tree rooted by using the closely related *Aphrodes bicincta* as an outgroup.

**Figure 5:**
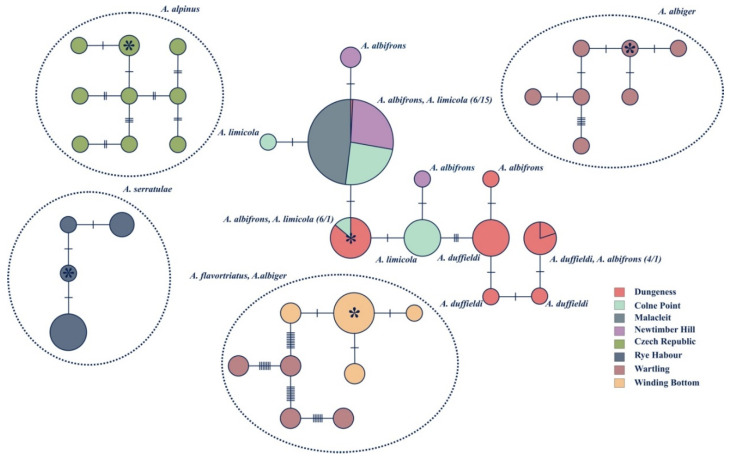
TCS network based on the mitochondrial CO1 gene. Each circle represents a unique haplotype, and the size of each circle is proportional to the number of samples. Cross-hatching along branches represents the number of mutational steps. (TCS only connects alleles with a 95% confidence limit, i.e., 10 steps). The colours represent localities, and the circles are labelled with the name of the haplotype as seen in Figure 2, Figure 3 and Figure 4. Stars indicate ancestral haplotypes.

**Figure 6:**
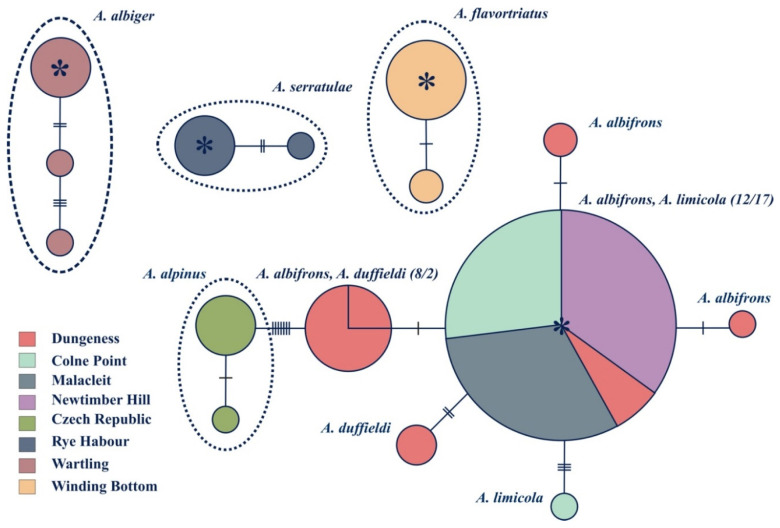
TCS network based on the mitochondrial 16S rRNA gene. Each circle represents a unique haplotype, and the size of each circle is proportional to the number of samples. Cross-hatching along branches represents the number of mutational steps. (TCS only connects alleles with a 95% confidence limit, i.e., 10 steps). The colours represent localities, and the circles are labelled with the name of the haplotype as seen in Figure 2, Figure 3 and Figure 4. Stars indicate ancestral haplotypes.

Three phylogenies were generated based on the CO1 gene (Figure 2), 16S rRNA gene (Figure 3) and the combined dataset including both the CO1 and 16S genes (Figure 4). Although sequencing was successful for 28S, this region failed to resolve closely related species as sequences were identical, hence these results are not shown. Phylogenies based on the CO1 gene and the 16S rRNA gene, in general, reflect the same topologies and mainly separated taxa monophyletically, with a few exceptions. Each tree separated the entire *Anoscopus* genus into two subgroups; one containing *A. albifrons*, *A. limicola* (Edwards, 1908), *A. duffieldi* and *A. alpinus* and the other *A. albiger*, *A. flavostriatus* (Donovan, 1799), *A. serratulae* and *A. histrionicus* (Fabricius, 1794), with strong support in each tree (posterior probabilities of 1) (Figure 2, Figure 3 and Figure 4). This was also reflected in the networks (Figure 5 and Figure 6). *Anoscopus albifrons* was clustered within both *A. limicola* and *A. duffieldi*, with two *A. albifrons* specimens grouping with *A. duffieldi* sequences and a number of *A. albifrons* and *A. limicola* specimens clustering together. There were shared haplotypes between *A. duffieldi* and *A. albifrons* and also between *A. limicola* and *A. albifrons*, indicating identical sequences across these taxa. The fourth species within this subgroup, *A. alpinus*, was clearly separated from the *A. duffieldi*, *A. limicola* and *A. albifrons* aggregate.

The other major clade that included *A. albiger*, *A. flavostriatus*, *A. serratulae* and *A. histrionicus* was resolved somewhat differently, but all species were clearly separated in the 16S tree (Figure 3), concatenated dataset (Figure 4) and network (Figure 6).

### 3.3. Population Level Analyses

Within-species sequence divergences were low in all species for the mitochondrial CO1 gene (divergences < 0.8%, CO1) except *A. albiger*, which harboured more within-species diversity (2.4%, CO1, Table 3). Genetic distances between *A. duffieldi*, *A. limicola* and *A. albifrons* were low, especially between *A. albifrons* and *A. limicola*, where a sequence divergence value of 0.05% was recorded for the CO1 gene and 0% for both the ribosomal 16S rRNA gene and the combined dataset. Sequence divergence values between *A. albifrons* and *A. duffieldi* were 0.35% (CO1), 0.15% (16S) and 0.35% (CO1 + 16S). Likewise, divergences between *A. limicola* and *A. duffieldi* were low at 0.8% (CO1), 0.15% (16S) and 0.55% (16S + CO1) (Table 5). These distances between species were well below the within-species genetic distances for *A. albiger*. However, it connects with the three-species aggregate when using 16S gene sequences (Figure 6). Haplotype diversity between species (groups identified in the TCS network) ranged from 0.38 to 1 and 0.11 to 0.50 for the CO1 and 16S, respectively. The suggested heterogeneity within *A. albiger* is mirrored by the high haplotype and nucleotide diversities recorded for this species (Table 4).

The geographic distribution of CO1 and 16S diversity within the genus *Anoscopus* are illustrated in Figure 5 and Figure 6, respectively. Using CO1 mitochondrial DNA sequences, the three well-defined lineages as indicated in the phylogenetic analyses (Figure 2, Figure 3 and Figure 4) were also geographically well defined (Figure 5). *Anoscopus alpinus* haplotypes were only recorded from the Czech Republic, *A. serratulae* haplotypes from Rye Habour and *A. albiger* haplotypes from Wartling. These networks could not be connected with 95% confidence, which indicates that these groups represent good biological species. Individuals representing *A. flavostriatus* and the remainder of *A. albiger* were connected with no shared haplotypes between these two species. All *A. flavostriatus* haplotypes were recorded from Winding Bottom and *A. albiger* individuals from Wartling. Individuals within *A. albiger* were separated from each other by up to 10 mutational steps, indicating higher levels of sequence variation within this species, and some of the *A. albiger* haplotypes could not be connected to each other and were closer to some of the *A. flavostriatus* haplotypes. However, there were no shared haplotypes between these two species. All those *A. flavostriatus* haplotypes were recorded from Winding Bottom and *A. albiger* individuals from Wartling. The last network included individuals of *A. albifrons*, *A. limicola* and *A. duffieldi*, with haplotypes being shared between species and localities. The results from the 16S rRNA networks (Figure 6) showed a similar pattern, with the exception that *A. alpinus* could be connected to the *A. albifrons*, *A. limicola* and *A. duffieldi* network. In addition, when using 16S rRNA sequences, *A. albiger* haplotypes were connected within one network.

## 4. Discussion

Species separation in *Aphrodes* and some of *Anoscopus* (e.g., *A. duffieldi* and *A. albifrons*) has hitherto been by aedeagus morphology. However, these characters alone have proved to be unreliable, although when combined with external morphometrics have been shown to improve species separation, at least for male *Aphrodes* [9,28]. It is likely that sexual vibrational communication signals in *Anoscopus* would provide additional evidence that may be diagnostic, as shown in *Aphrodes* [28], but this requires specialist equipment and expertise that is not widely available. Some Canadian *Anoscopus* spp. have been barcoded previously based on specimens collected in Canada and Corsica [59,60], but here we used DNA barcoding for the first time to separate all the known species of *Anoscopus* in the UK, with unexpected results.

The phylogenetic trees showed a major, deep division within the *Anoscopus* genus, with one subgroup comprising *A. albifrons*, *A. limicola*, *A. duffieldi* and *A. alpinus* (*albifrons* subgroup) and the other including *A. albiger*, *A. flavostriatus*, *A. serratulae* and *A. histrionicus* (*albiger* subgroup). There appear to be no obvious morphological differences between these subgroups that might warrant further taxonomic recognition. However, our analysis lacked several additional *Anoscopus* species and subspecies described from continental Europe, Asia and the Canary Islands [20,61], which would be needed to fully understand the phylogeny of the genus. However, it should be remembered that our aim here was primarily to separate species from the UK and determine the status of *A. duffieldi*, and not to generate a complete phylogeny.

### 4.1. Anoscopus duffieldi and Related Taxa (Albifrons Subgroup)

The phylogenetic trees (Figure 2, Figure 3 and Figure 4) and analysis of genetic distances (Table 3 and Table 5) clearly show that *A. duffieldi* is not conspecific with *A. alpinus* (based on specimens from the Czech Republic), as proposed by Le Quesne [23]. Besides differences in mitochondrial DNA sequences, both taxa also differ in habitat preferences and have allopatric distributions. *Anoscopus duffieldi* has only been recorded from vegetated coastal shingle at Dungeness, Kent [21], while *Anoscopus alpinus* is restricted to heaths, bogs and subalpine grasslands at high elevations (between 880 and 2970 m a.s.l.) of central and eastern European mountains: the Alps, Hercynian mountains, the Balkans and probably also the Carpathians [12,20,62]. However, we cannot eliminate the possibility that *A. duffieldi* is synonymous with another continental species, *A. assimilis*, to which it is also morphologically similar [12,24,27], because the latter species was missing in our molecular dataset. *Anoscopus assimilis* has been reported from meadows, pastures and undergrowth of mixed forests at low to montane elevations of the western Mediterranean region, and its distribution seems to extend in western France as far north as to Brittany [20,22,63]. We were able to download and examine two identical CO1 sequences thought to be *A. assimilis* collected in Corsica [60] (GenBank accession numbers MK816310 and MK188564), but these were acquired from females and hence, the authors acknowledge, impossible to accurately identify morphologically. They shared an identical haplotype with both *A. duffieldi* and *A. albifrons* (h1 in Figure 2).

*Anoscopus duffieldi* was found sympatrically with *A. albifrons* at Dungeness. *Anoscopus duffieldi* cluster together in all trees (Figure 5 and Figure 6). However, in the same cluster with *A. duffieldi* are specimens of *A. albifrons*, including a haplotype that is found in both species. Possible reasons for this include misidentification caused by intermediate aedeagal characters. Alternatively, there may be uni-directional hybridisation where male individuals of *A. albifrons* mate with female individuals of *A. duffieldi* to produce morphologically *A. albifrons* individuals but with *A. duffieldi* mitochondria. Hybridisation may also result in mixed characters [20], hindering correct identification. The term ‘hybridisation’ of course is not entirely correct for crosses between taxa that are subspecies or ecotypes.

*Anoscopus duffieldi* specimens are also closely related to a mixed cluster of *A. albifrons* and *A. limicola*. For the mitochondrial genes studied, there is no evidence that these are separate species. Sequence divergence values between *A. albifrons* and *A. limicola* was estimated at 0–0.05% (Table 5). There were several shared haplotypes between these two taxa, suggesting that they are one interbreeding population. Interestingly, a shared haplotype between *A. limicola* and *A. albifrons* is found at both Newtimber Hill on the south coast and Malacleit in the Outer Hebrides (morphologically identified as *A. albifrons* at Newtimber Hill and *A. limicola* in Malacleit). Divergence values between *A. duffieldi* and both *A. albifrons* and *A. limicola* were lower than would be expected for different species, and much lower than between other species of *Anoscopus* (Table 5). *Anoscopus albifrons* and *A. limicola* differ mainly in the general size, subtle details of aedeagus shape and ecology. While the former is a quite eurytopic and widely distributed grassland species, the latter has been considered to be a salt marshes specialist, particularly on the grass species *Puccinellia maritima*, and restricted to western European coasts [12,20,33,63].

### 4.2. Other Anoscopus Species (Albiger Subgroup)

All of the other species separated well, forming monophyletic groups with low intraspecific genetic diversity, with the exception of *A. albiger*. Some haplotypes of this highly genetically diverse species show affinities with *A. flavostriatus* in the CO1 tree (Figure 2), but these two species are resolved into monophyletic sister groups in the 16S and combined (CO1 + 16S) trees (Figure 3 and Figure 4). All the *A. albiger* specimens came from the same location (Wartling), yet each of the individuals harboured a unique haplotype. This strongly suggests high levels of genetic diversity within this species. *Anoscopus albiger* was clearly different from all the other *Anoscopus* in having far greater intraspecific diversity (e.g., Table 3, 2.4% at CO1, compared with <0.8% for all other groups). A possible explanation for this intraspecific diversity within *A*. *albiger* is that it has had a very different history in the UK compared with the other *Anoscopus* species. One possibility is that this is a relict species that managed to survive in the UK through the last ice age, retaining high levels of genetic diversity. The low levels of genetic diversity shown in all of the other *Anoscopus* species may indicate that they went through genetic bottlenecks during post-glacial recolonization. More sampling from other parts of the UK, Ireland and continental Europe may help to resolve this question.

## 5. Conclusions

**Figure 7:**
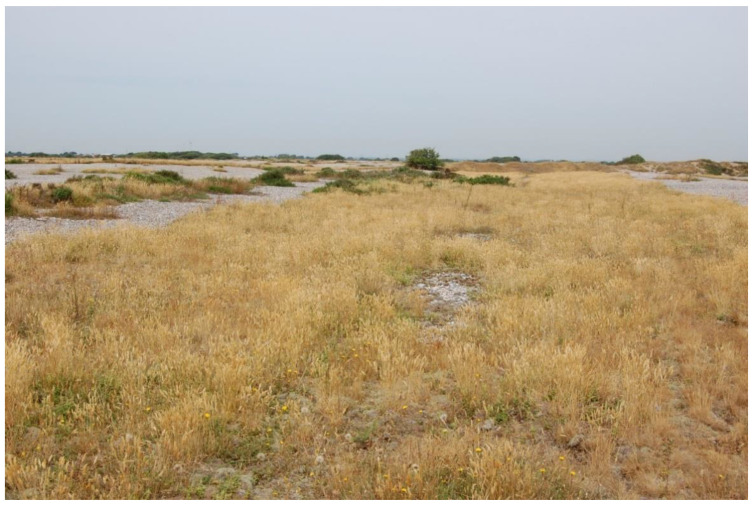
Vegetated shingle at Dungeness dominated by the grass *Anthoxanthum odoratum*. Dungeness is the only known site where *Anoscopus duffieldi* has been recorded. Photo taken by Alan Stewart.

Five out of eight *Anoscopus* taxa studied were clearly separated through mtDNA barcoding, and based on both morphological and molecular evidence, they represent distinct species. For the remaining three taxa (*A. duffieldi*, *A. albifrons* and *A. limicola*), there is little support for their status as separate species based on our molecular evidence. Pairwise genetic distances among these three taxa were very low, ranging from 0% to 0.55% (CO1 + 16S). In contrast, pairwise comparisons between all other species, and between these other species and *A. duffieldi*, *A. albifrons* and *A. limicola*, ranged from 4.05% to 10.45%. There is little support therefore for *A. duffieldi* as a separate species. However, specimens of *A. duffieldi* did cluster together in the trees, so it would be prudent to protect this population until other evidence is forthcoming and in the meantime treat *A. duffieldi* provisionally as a subspecies with a unique morphotype or simply a different ecotype or possibly host race of *A. albifrons*. More research would be needed to establish which term would be most appropriate. The only habitat and site on which they have been found is dry shingle, dominated by the grass *Anthoxanthum odoratum* L. (Figure 7). It might be appropriate to attribute a similar status (subspecies, ecotype or host race of *A. albifrons*) to *A. limicola*. All the *A. limicola* specimens came from saltmarsh dominated by *Puccinellia maritima* (Hudson), which is consistent with previously published data on the ecology of this taxon [12]. *Anoscopus albifrons* has been considered a generalist species found in a wide range of habitats. Guglielmino and Bückle [20] recently described another distinct morphotype as a subspecies from southern Europe, *A. albifrons mappus*. *Anoscopus albifrons* in its broad sense may turn out to represent a single polymorphic species or a complex of incipient species undergoing a process of speciation, but this suggestion clearly requires more research.

Further work could include sequencing of nuclear genes that are less conserved than 28S. This might shed light on possible cases of hybridisation. Other studies have successfully amplified the nuclear protein-coding genes Histone 3 and Wingless from leafhoppers to resolve relationships between species [64]. The hypervariable mitochondrial D-loop region could possibly resolve the relationships further. Microsatellites have been developed for the closely related *Aphrodes* [65], and these should be tested to see whether they work on *Anoscopus*. If not, more specific microsatellites could be developed. Another option is to include other characteristics such as vibrational signals [9] and ecological information such as habitat and food plant data [20]. Future studies should also aim to include material of *A. assimilis* and other continental taxa which are missing from our analysis.

Britain has few endemic species, and these have historically been afforded priority status by conservationists within the Biodiversity Acton Plan process [32]. Significantly, in the context of this study, however, Britain has several distinct subspecific varieties or forms of invertebrates that are endemic, often differing from their continental counterparts ecologically as well as morphologically. Furthermore, and probably because of its unique habitat for invertebrates (Figure 7) ([66]), Dungeness harbours a significant number of these endemic variants [32], of which *A. duffieldi* may be one.

## Figures and Tables

**Table 1:** Collection site, year and storage method for the *Anoscopus* specimens sequenced.

*Anoscopus* Species	Collection Site	Ordnance Survey ^+^	Co-Ordinates (N, E)	Year Collected	Storage
*albifrons*	Dungeness, Kent	TR076190	50.933074, 0.95310771	2013	100% Ethanol *
	Newtimber Hill, Sussex	TQ268119	50.892674, −0.19846861	2015	100% Ethanol
*albiger*	Wartling, Sussex	TQ666085	50.852027, 0.36542503	2014/15	Dried/100% Ethanol
*alpinus*	Mt Kralicky Sneznik, Czech Rep.		50.206401, 16.849404	2015	100% Ethanol
*duffieldi*	Dungeness, Kent	TR076190	50.933074, 0.95310771	2014/15	100% Ethanol
*flavostriatus*	Winding Bottom, Sussex	TQ191087	50.865548, −0.30893968	2013	Dried
				2014/15	100% Ethanol
*limicola*	Colne Point, Essex	TM108124	51.770566, 1.0538762	2011	100% Ethanol *
	Malacleit, Outer Hebrides	NF790730	56.632199, −7.3805002	2012	Dried
*serratulae*	Rye Harbour, Sussex	TQ931192	50.939904, 0.74711850	2015	100% Ethanol
*histrionicus*	Merthyr Common, Wales	SO071058	51.743173, −3.3469341	2015	Ethanol ^§^

^+^ Ordinance Survey is the National mapping agency for Great Britain and is widely used for determining co-ordinates. * specimens were frozen prior to transfer into 100% ethanol. ^§^ specimen killed with ethyl acetate, frozen and dried prior to transfer into molecular-grade ethanol.

**Table 2:** Forward (top) and reverse (bottom) primer names, sequences (5′ to 3′) and reference for each primer pair for the mitochondrial CO1 (Cytochrome Oxidase 1) gene, the mitochondrial ribosomal gene16S rRNA and the nuclear 28S ribosomal RNA gene (rRNA).

Locus	Primer Name	Primer Sequence (5′–3′)	Reference
CO1	LCO1490	GGTCAACAAATCATAAAGATATTGG	Folmer et al., 1994 [40]
	HCO2198	TAAACTTCAGGGTGACCAAAAAATCA	
16S	LR-J-12887	CCGGTYTGAACTCARATCAWGT	Fu et al., 2014 [41]
	LR-N-13398	CTGTTTAWCAAAAACATTTC	
28S	28SP	AGTCGKGTTGCTTGAKAGTGCAG	Zahniser, 2008 [43]
	28SM2	TTCGGGTCCCAACGTGTACG	
	28SII’(F)	GGGACCCGTCTTGAAACAC	Dietrich et al., 2001 [38]
	28SII’(R)	ACCCTCCTACTCGTCAAGG	

**Table 3:** Within-group (intraspecific) mean pairwise distances (d) with corresponding standard errors (generated by MEGA v6.0; [56] for CO1and 16S, for *Anoscopus* species. *Anoscopus histrionicus* was not included as there was only one individual available. *d* = divergence, S.E. = standard error.

Species	CO1		16S	
	*d* (%)	S.E. (%)	*d* (%)	S.E. (%)
*albifrons*	0.8	0.2	0.4	0.2
*albiger*	2.4	0.4	0.7	0.3
*alpinus*	0.6	0.2	0.3	0.3
*duffieldi*	0.3	0.2	0.3	0.3
*flavostriatus*	0.3	0.2	0.3	0.3
*limicola*	0.3	0.1	0.8	0.5
*serratulae*	0.4	0.2	0.5	0.3

**Table 4:** Mitochondrial cytochrome oxidase 1 and 16S rRNA per cent haplotype and nucleotide diversity as identified by the network and phylogenetic analyses within *Anoscopus*.

Species	CO1		16S rRNA	
	Haplotype Diversity	Nucleotide Diversity	Haplotype Diversity	Nucleotide Diversity
*A. albifrons*	0.81	0.004	0.5	0.001
*A. albiger*	1.00	0.024	0.46	0.003
*A. alpinus*	0.98	0.006	0.29	0.0007
*A. duffieldi*	0.68	0.003	0.44	0.001
*A. flavostriatus*	0.65	0.001	0.33	0.0009
*A. limicola*	0.38	0.001	0.11	0.0009
*A. serratulae*	0.81	0.004	0.29	0.001

**Table 5:** Mean pairwise distances (%) (corrected for intra-specific distances) between *Anoscopus* taxa for CO1 and 16S in bold below each diagonal with standard error values (%) above (generated by MEGA v6.0; [56] Tamura et al., 2013). *Anoscopus histrionicus* was not included as there was only one individual available.

Loci and Species Name	Mean Genetic Distance/Standard Error (%)					
	1	2	3	4	5	6
CO1						
1. *albifrons*		1	0.9	0.3	1.2	0.2
2. *albiger*	**10.2**		1.2	1	0.7	1.1
3. *alpinus*	**6.2**	**10.6**		0.9	1.3	0.9
4. *duffieldi*	**0.35**	**10.05**	**6.35**		1.2	0.4
5. *flavostriatus*	**13.25**	**4.15**	**13.25**	**13.1**		1.2
6. *limicola*	**0.05**	**10.65**	**6.55**	**0.8**	**13.7**	
7. *serratulae*	**11.4**	**6.7**	**11.7**	**11.15**	**8.25**	**11.85**
16S						
1. *albifrons*		1.2	0.7	0.2	1	0.1
2. *albiger*	**5.25**		1.1	1.2	0.8	1.2
3. *alpinus*	**1.95**	**5.2**		0.6	1	0.6
4. *duffieldi*	**0.15**	**5.2**	**1.9**		1	0.3
5. *flavostriatus*	**5.25**	**3.6**	**4.9**	**5.2**		1
6. *limicola*	**0**	**5.35**	**2.15**	**0.15**	**5.35**	
7. *serratulae*	**5.05**	**4.1**	**5.2**	**4.9**	**2.6**	**5.05**

## Data Availability

GenBank accession numbers for all *Anoscopus* species and haplotypes, plus haplotypes shared between species, plus the 16S sequence for *A. bicincta*: *Anoscopus albifrons* COI MW204907, MW204910, MW204913 16S MW228389, MW228390; *Anoscopus albiger* COI MW204884-MW204894 16S MW228379-MW228381; *Anoscopus alpinus* COI MW204914-MW204922 16S MW228393, MW228394; *Anoscopus duffieldi* COI MW204904-MW204906 16S MW228392; *Anoscopus flavostriatus* COI MW204895-MW204898 16S MW228382, MW228383; *Anoscopus histrionicus* COI MW204923 16S MW228386; *Anoscopus limicola* COI MW204911 16S MW228391; *Anoscopus serratulae* COI MW204899-MW204902 16S W228384, MW228385; *Anoscopus limicola/albifrons* haplotype COI MW204908, MW204909, MW204912 16S MW228387; *Anoscopus duffieldi/albifrons* haplotype COI MW204903 16S MW228388; *Aphrodes bicincta* 16S MW228395.

## References

[1] Austin J.J., Melville J. (2006). Incorporating historical museum specimens into molecular systematic and conservation genetics research. Mol. Ecol. Notes.

[2] Hebert P.D.N., Gregory T.R. (2005). The promise of DNA barcoding for taxonomy. Syst. Biol..

[3] Dexter K.G., Pennington T.D., Cunningham C.W. (2010). Using DNA to assess errors in tropical tree identifications: How often are ecologists wrong and when does it matter?. Ecol. Monogr..

[4] Lis B., Lis B., Ziaja D.J. (2016). In BOLD we trust? A commentary on the reliability of specimen identification for DNA barcoding: A case study on burrower bugs (Hemiptera: Heteroptera: Cydnidae). Zootaxa.

[5] Bluemel J.K., King R., Virant-Doberlet M., Symondson W.O.C. (2011). Primers for identification of type and other archived specimens of *Aphrodes* leafhoppers (Hemiptera, Cicadellidae). Mol. Ecol. Resour..

[6] Seabra S.G., Pina-Martins F., Marabuto E., Yurtsever S., Halkka O., Quartau J.A., Paulo O.S. (2010). Molecular phylogeny and DNA barcoding in the meadow-spittlebug *Philaenus spumarius* (Hemiptera, Cercopidae) and its related species. Mol. Phylogenet. Evol..

[7] Aly S.M. (2014). Reliability of long vs short COI markers in identification of forensically important flies. Croat. Med. J..

[8] De Mandal S., Chhakchhuak L., Gurusubramanian G., Kumar N.S. (2014). Mitochondrial markers for identification and phylogenetic studies in insects—A review. DNA Barcodes.

[9] Bluemel J.K., Derlink M., Pavlovčič P., Russo I.-R.M., King R.A., Corbett E., Sherrard-Smith E., Blejec A., Wilson M.R., Stewart A.J.A. (2014). Integrating vibrational signals, mitochondrial DNA and morphology for species determination in the genus *Aphrodes* (Hemiptera: Cicadellidae). Syst. Entomol..

[10] Kirby P., Stewart A.J., Wilson M.R., Hawksworth D.L. (2001). True bugs, leaf-and planthoppers, and their allies. The Changing Wildlife of Great Britain and Ireland.

[11] Forero D. (2008). The systematics of the Hemiptera. Rev. Colomb. Entomol..

[12] Nickel H. (2003). The Leafhoppers and Planthoppers of Germany (Hemiptera: Auchenorrhyncha): Patterns and Strategies in a Highly Diverse Group of Phytophagous Insects.

[13] Bartlett C.R., Deitz L.L., Dmitriev D.A., Sanborn A.F., Soulier-Perkins A., Wallace M.S., Foottit R.G., Adler P.H. (2018). The diversity of the true hoppers (Hemiptera: Auchenorrhyncha). Insect Biodiversity: Science and Society.

[14] Biedermann R., Achtziger R., Nickel H., Stewart A.J.A. (2005). Conservation of grassland leafhoppers: A brief review. J. Insect Conserv..

[15] Zahniser J.N., Dietrich C.H. (2008). Phylogeny of the leafhopper subfamily Deltocephalinae (Insecta: Auchenorrhyncha: Cicadellidae) and related subfamilies based on morphology. Syst. Biodivers..

[16] Dietrich C.H., Chang C.-I., Lee C.-Y., Hsien-Tzung Shih H.-T. (2013). Overview of the phylogeny, taxonomy and diversity of the leafhopper (Hemiptera: Auchenorrhyncha: Cicadomorpha: Membracoidea: Cicadellidae) vectors of plant pathogens. Proceedings of the 2013 International Symposium on Insect Vectors and Insect-Borne Diseases, Taichung, Taiwan, August 2013.

[17] Rothenbücher J., Schaefer M. (2005). Conservation of leafhoppers in floodplain grasslands—Trade-off between diversity and naturalness in a northern German national park. J. Insect Cons..

[18] Schuch S., Wesche K., Schaefer M. (2012). Long-term decline in the abundance of leafhoppers and planthoppers (Auchenorrhyncha) in Central European protected dry grasslands. Biol. Conserv..

[19] Endrestøl A., Elven H. (2009). Two species of Aphrodinae (Hemiptera, Cicadellidae) new to the Norwegian fauna. Norweg. J. Entomol..

[20] Guglielmino A., Bückle C. (2015). Revision of Errhomeninae and Aphrodinae (Hemiptera, Cicadomorpha) in Italy with remarks on their variability and distribution in adjacent regions and description of three new taxa. Zootaxa.

[21] Wilson M.R., Stewart A., Biedermann R., Nickel H., Niedringhaus R. (2015). The Planthoppers and Leafhoppers of Britain and Ireland. Identification Keys to All Families and Genera and all British and Irish Species not Recorded from Germany. Cicadina, Supplement 2.

[22] Ribaut H. (1952). Homoptères Auchénorhinques. II. (Jassidae).

[23] Le Quesne W.J. (1965). Hemiptera (Cicadomorpha), excluding Deltocephalinae and Typhlocybinae. Handbooks for the Identification of British Insects.

[24] Hamilton K.G.A. (1975). A review of the northern hemisphere aphrodina (Rhynchota: Homoptera: Cicadellidae), with special reference to the nearctic fauna. Can. Entomol..

[25] Biedermann R., Niedringhaus R. (2009). The Plant- and Leafhoppers of Germany: Identification Key to All Species.

[26] Barnett D.E. (1976). Some new preparation techniques used in leafhopper identification. Fla. Entomol..

[27] Remane R., Fröhlich W. (1994). Beiträge zur Chorologie einiger Zikaden-Arten (Homoptera Auchenorrhyncha) in der Westpaläarktis. Marbg. Entomol. Publik..

[28] Tishechkin D.Y. (2017). On the taxonomy and distribution of Aphrodes bicincta (Schrank, 1776) species group (Homoptera: Auchenorrhyncha: Cicadellidae: Aphrodinae) in Eastern Palaearctic. Zootaxa.

[29] Duffield C.A.W. (1963). The genus aphrodes (Homoptera; Auchenorhyncha). Trans. Kent Field Club.

[30] Le Quesne W.J. (1964). Some taxonomic changes and additions in the British Cicadellidae (Hemiptera) including a new species and subspecies. Proc. R. Entomol. Soc. B.

[31] Nast J. (1992). Palaearctic Auchenorrhyncha (Homoptera) an Annotated Check List.

[32] Key R.S., Drake C.M., Sheppard D.A. (2000). Conservation of Invertebrates in England: A Review and Framework.

[33] Ossiannilsson F. (1981). The auchenorrhyncha (homoptera) of fennoscandia and Denmark. Fauna Entomologica Scandinavica—Part 2.

[34] Pentinsaari M., Salmela H., Mutanen M., Roslin T. (2016). Molecular evolution of a widely-adopted taxonomic marker (COI) across the animal tree of life. Sci. Rep..

[35] Percy D.M. (2007). Making the most of your host: The Metrosideros-feeding psyllids (Hemiptera, Psylloidea) of the Hawaiian Islands. ZooKeys.

[36] Hebert P.D., Ratnasingham S., De Waard J.R. (2003). Barcoding animal life: Cytochrome c oxidase subunit 1 divergences among closely related species. Proc. R. Soc. B Biol. Sci..

[37] Nei M., Li W.H. (1979). Mathematical model for studying genetic variation in terms of restriction endonucleases. Proc. Natl. Acad. Sci. USA.

[38] Dietrich C., Rakitov R., Holmes J., Black W. (2001). Phylogeny of the major lineages of membracoidea (Insecta: Hemiptera: Cicadomorpha) based on 28S rDNA sequences. Mol. Phylogenet. Evol..

[39] Dai R.H., Chen X.X., Li Z.Z. (2008). Phylogeny of Deltocephalinae (Hemiptera: Cicadellidae) from China based on partial 16S rDNA and 28S rDNA D2 sequences combined with morphological characters. Acta Entomol. Sin..

[40] Folmer O., Black M., Hoeh W., Lutz R., Vrijenhoek R. (1994). DNA primers for amplification of mitochondrial cytochrome c oxidase subunit I from diverse metazoan invertebrates. Mol. Mar. Biol. Biotechnol..

[41] Fu J.-Y., Han B.-Y., Xiao Q. (2014). Mitochondrial COI and 16sRNA evidence for a single species hypothesis of *E. vitis*, *J. formosana* and *E. onukii* in East Asia. PLoS ONE.

[42] Hillis D.M., Dixon M.T. (1991). Ribosomal DNA: Molecular evolution and phylogenetic inference. Q. Rev. Biol..

[43] Zahniser J.N. (2008). Systematics of the Leafhopper Subfamily Deltocephalinae (Hemiptera: Cicadellidae) and the Tribe Chiasmini: Phylogeny, Classification, and Biogeography. Ph.D Thesis.

[44] Larkin M., Blackshields G., Brown N., Chenna R., Mcgettigan P., McWilliam H., Valentin F., Wallace I.M., Wilm A., Lopez R. (2007). Clustal W and Clustal X version 2.0. Bioinformatics.

[45] Guindon S., Gascuel O. (2003). A simple, fast, and accurate algorithm to estimate large phylogenies by maximum likelihood. Syst. Biol..

[46] Darriba D., Taboada G.L., Doallo R., Posada D. (2012). jModelTest 2: More models, new heuristics and parallel computing. Nat. Methods.

[47] Yang Z., Goldman N., Friday A. (1994). Comparison of models from nucleotide substitution used in maximum likelihood phylogenetic estimation. Mol. Biol. Evol..

[48] Yang Z. (1996). Among-site rate variation and its impact on phylogenetic analyses. Trends Ecol. Evol..

[49] Ronquist F., Teslenko M., Van Der Mark P., Ayres D.L., Darling A., Höhna S., Larget B., Liu L., Suchard M.A., Huelsenbeck J.P. (2012). MrBayes 3.2: Efficient bayesian phylogenetic inference and model choice across a large model space. Syst. Biol..

[50] Rambaut A., Suchard M., Xie W., Drummond A. (2014). *Tracer v. 1.6*; Institute of Evolutionary Biology, University of Edinburgh. https://github.com/beast-dev/tracer/releases/tag/v1.6.

[51] Rambaut A. (2014). *FigTree 1.4. 2*; University of Edinburgh: Edinburgh, Scotland. http://tree.bio.ed.ac.uk/software/figtree/.

[52] Nei M., Tajima F. (1981). Genetic drift and estimation of effective population size. Genetics.

[53] Nei M. (1987). Molecular Evolutionary Genetics.

[54] Rozas J., Rozas R. (1999). DnaSP version 3: An integrated program for molecular population genetics and molecular evolution analysis. Bioinformatics.

[55] Librado P., Rozas J. (2009). DnaSP v5: A software for comprehensive analysis of DNA polymorphism data. Bioinformatics.

[56] Tamura K., Stecher G., Peterson D., Filipski A., Kumar S. (2013). MEGA6: Molecular Evolutionary Genetics Analysis version 6.0. Mol. Biol. Evol..

[57] Clement M., Posada D., Crandall K.A. (2000). TCS: A computer program to estimate gene genealogies. Mol. Ecol..

[58] Avise J.C. (2000). Phylogeography: The History and Formation of Species.

[59] Gwiazdowski R.A., Foottit R.G., Maw H.E.L., Hebert P.D.N. (2015). The Hemiptera (Insecta) of Canada: Constructing a reference library of DNA barcodes. PLoS ONE.

[60] Albre J., Gibernau M. (2019). Diversity and temporal variations of the Hemiptera Auchenorrhyncha fauna in the Ajaccio region (France, Corsica). Ann. Soc. Entomol. Fr..

[61] Lindberg H. (1954). Hemiptera insularum canariensium. systematik, ökologie und verbreitung der kanarischen heteropteren und cicadinen. Comment. Biol..

[62] Malenovský I. (2013). New records of Auchenorrhyncha (Hemiptera) for the Czech Republic. Acta Musei Morav. Scient. Boil..

[63] Della Giustina W. (1989). Homoptères Cicadellidae. Compléments aux ouvrages d’Henri Ribaut. Faune de France, 73.

[64] Bennett G.M., O’Grady P.M. (2011). Review of the native Hawaiian leafhopper genus Nesophrosyne (Hemiptera: Cicadellidae: Deltocephalinae) with description of eight new species associated with Broussaisia arguta (Hydrangeaceae). Zootaxa.

[65] Derlink M., Pipan B., Pavlovčič P., Jones L., Meglič V., Symondson W.O.C., Virant-Doberlet M. (2014). Characterization of eleven polymorphic microsatellite markers for leafhoppers of the genus *Aphrodes* (Hemiptera: Cicadellidae). Conserv. Genet. Resour..

[66] Morris R.K.A., Parsons M.S. (1992). A survey of invertebrate communities on the shingle of Dungeness, Rye Harbour and Orford Ness. JNCC Report 77.

